# Impact of intraoperative hypothermia on the recovery period of anesthesia in elderly patients undergoing abdominal surgery

**DOI:** 10.1186/s12871-024-02509-6

**Published:** 2024-04-01

**Authors:** Lu Yin, Heng Wang, Xiaorong Yin, Xiuying Hu

**Affiliations:** 1 Department of Anesthesiology, West China Hospital, West China School of Nursing, Sichuan University, Chengdu, Sichuan Province 610041 People’s Republic of China; 2 Innovation Center of Nursing Research and Nursing Key Laboratory of Sichuan Province, West China Hospital, West China School of Nursing, Sichuan University, Chengdu, 610041 People’s Republic of China

**Keywords:** Elderly patients, Abdominal surgery, Hypothermia, Anesthesia recovery

## Abstract

**Background:**

This study aimed to investigate the impact of intraoperative hypothermia on the recovery period of anesthesia in elderly patients undergoing abdominal surgery.

**Methods:**

A prospective observational study was conducted based on inclusion and exclusion criteria. A total of 384 elderly patients undergoing abdominal surgery under general anesthesia were enrolled in a grade A tertiary hospital in Chengdu, Sichuan Province from October 2021 and October 2022. After anesthesia induction, inflatable warming blankets were routinely used for active heat preservation, and nasopharyngeal temperature was monitored to observe the occurrence of intraoperative hypothermia. Patients were divided into hypothermia group and nonhypothermia group according to whether hypothermia occurred during the operation. Anesthesia recovery time and the incidence of adverse events or unwanted events during anesthesia recovery between the two groups were compared.

**Results:**

The numbers (percentage) of 384 patients who underwent abdominal surgery developed intraoperative hypothermia occurred in 240 (62.5%) patients, all of whom had mild hypothermia. There were statistically significant differences between mild hypothermia after active warming and nonhypothermia in the occurrence of shivering (χ2 = 5.197, *P* = 0.023) and anesthesia recovery time (Z = -2.269, *P* = 0.02) in elderly patients undergoing abdominal surgery during anesthesia recovery, and there were no statistically significant differences in hypoxemia, nausea or vomiting, hypertension, hypokalemia, hypocalcemia, analgesic drug use,postoperative wound infection or postoperative hospitalization days.

**Conclusions:**

The incidence of intraoperative mild hypothermia after active warming was high in elderly patients who underwent abdominal surgery. Mild hypothermia increased the incidence of shivering and prolonged anesthesia recovery time in elderly patients undergoing abdominal surgery.

## Background

Intraoperative hypothermia is one of the most common problems during anesthesia. It refers to a patient’s core body temperature falling below 36℃ from the start of anesthesia until the completion of the surgical procedure [1], but does not include therapeutic hypothermia. According to the different severities of hypothermia, it can be divided into mild hypothermia (34℃-35.9℃), moderate hypothermia (32℃-33.9℃), and severe hypothermia (< 32℃) [1]. Based on recent research findings, the incidence of intraoperative hypothermia in patients undergoing general anesthesia ranges from 50 to 70% [2]. However, it is crucial to note that elderly patients undergoing abdominal surgery are particularly susceptible to intraoperative hypothermia compared to younger counterparts. This heightened vulnerability can be attributed to degenerative changes in the body, low metabolic rates, reduced reserve function, poor tolerance, and inadequate compensatory abilities [3]. Consequently, these physiological characteristics contribute to a more rapid decline in core body temperature during surgery, particularly when stimulated by the surgical source. According to domestic and foreign studies,the incidence of intraoperative hypothermia in elderly patients undergoing abdominal surgery ranges from 39.26% to 85.42% [4, 5]. Once intraoperative hypothermia occurs, it may lead to various adverse outcomes in elderly patients undergoing abdominal surgery [6, 7], such as inducing cardiovascular events, compromising wound healing, increasing surgical site infection rate, impairing coagulation function and increasing postoperative oxygen consumption. However, There is relatively limited research on the impact of intraoperative mild hypothermia on anesthesia recovery period.Therefore, this study aims to evaluate the impact of intraoperative mild hypothermia on the anesthesia recovery period in elderly patients undergoing abdominal surgery to provide a theoretical basis for nursing intervention.

## Materials and methods

### Patients and ethics

This observational study received approval from the Clinical trial and Biomedical Ethics Committee, West China Hospital, Sichuan University, Chengdu, China. Elderly patients who underwent abdominal surgery at our institution between October 2021 and October 2022 were enrolled in this study.

### Inclusion and exclusion criteria

The inclusion criteria were as follows: (1) age ≥ 60 years; (2) abdominal surgical categories, including gastrointestinal surgery, liver surgery, pancreatic surgery, biliary surgery, and splenic surgery; (3) preoperative baseline body temperature between 36 °C and 37 °C; (4) provided informed consent and willingly participated in this study.

The exclusion criteria were as follows: (1) Patients with contraindications for monitoring nasopharyngeal temperature, including anatomical abnormalities in the nasopharynx or coagulation disorders (platelet count < 100 * 10^9, international normalized ratio > 1.2, activated partial thromboplastin time > 40 s; meeting any of these criteria indicates a coagulation disorder [8]); (2) patients with preexisting conditions that result in abnormal body temperature, such as hyperthyroidism, hypothyroidism, infectious diseases, etc.

### Core temperature measurement

The operating room temperature was set at 22–24 ℃. After successfully inserting the endotracheal tube for the patient, a conventional inflatable warming blanket was utilized for active warming, with the heaters set at a temperature of 38℃. Nasopharyngeal temperature testing is used for all patients, and during the perianesthesia period, we performed regular temperature recording of the patient's nasopharynx.The temperature monitoring probe was adequately lubricated with petroleum jelly and then inserted into the patient's nasal cavity until reaching the nasopharynx, with an insertion depth from the patient's nasal tip to the earlobe. The temperature probe was secured with adhesive tape, and the probe cable was connected to the temperature monitoring module of the monitor. The temperature of the patient's nasopharynx was continuously and dynamically monitored. The temperature probe was also removed after the patient had the endotracheal tube removed. The patients' nasopharyngeal temperatures were recorded at the end of induction of anesthesia (T0), 0.5h after anesthesia (T1), 1h after anesthesia (T2), 1.5h after anesthesia (T3), 2h after anesthesia (T4), 2.5h after anesthesia (T5), 3h after anesthesia (T6), 3.5h after anesthesia (T7), 4h after anesthesia (T8), 4.5h after anesthesia (T9), 5h after anesthesia (T10), nasopharyngeal temperature at 5.5h after anesthesia (T11), 6h after anesthesia (T12).

### Anesthesia

All elderly patients undergoing abdominal surgery received invasive arterial blood pressure monitoring through radial artery cannulation after anesthesia induction. Bispectral index (BIS) monitoring was used to assess the depth of anesthesia. During the surgery, total intravenous anesthesia was administered using the following doses: midazolam 0.05 mg/kg, droperidol 0.01 mg/kg, sufentanil 0.3–0.4 µg/kg, cisatracurium 0.15–0.2 mg/kg, and propofol 1.5–2 mg/kg for anesthesia induction. A size 7 or 7.5 endotracheal tube was inserted under direct laryngoscopy guidance. Anesthesia was maintained with sevoflurane (1%-2%), propofol (2–4 mg/kg/h), and remifentanil (0.1–0.2 µg/kg/min), with intermittent supplementation of sufentanil (0.1 µg/kg) and cisatracurium (0.05–0.1 mg/kg). The BIS value was maintained between 40 and 60. The irrigation fluid was warmed to 37℃.

### Outcome measures

The primary outcome was inadvertent intraoperative hypothermia, defined as core temperature < 36 °C for 5 min during the intraoperative period. Body temperature was recorded via an esophageal probe during surgery. Patients with nasal-pharyngeal temperature < 36℃ and duration > 5 min were classified as the hypothermia group, while patients with nasal-pharyngeal temperature ≥ 36℃ or nasal-pharyngeal temperature < 36℃ but duration < 5 min were classified as the nonhypothermia group. Essential characteristics of all patients, such as age, sex,body mass index(BMI), duration of surgery,American Society of Anesthesiologists (ASA) grade,Total amount of fluid administration,urine output,types of surgery,etc. were collected. Comparison of anesthetic recovery time and adverse events or unwanted events during anesthetic rcovery (shivering, hypoxemia, nausea or vomiting, analgesic drug use, hypokalemia, hypocalcemia), incidence of postoperative wound infection, and postoperative hospitalization days in two patient groups.Shivering was divided into 5 grade,including:grade 0:No shivering; grade 1:One or more of: piloerection, peripheral vasoconstriction,peripheral cyanosis without other cause, but without visible muscular activity; grade 2: Visible muscular activity confined to one muscle group; grade 3: visible muscular activity in more than one muscle group; grade 4:Gross muscular activity involving entire body.Grade 0 indicated no shivering, and grade 1 and above indicated shivering [9]. Hypoxemia was defined as a pulse oximeter saturation of less than 92%.Anesthesia recovery time was defined as the duration from patient admission to discharge from the post-anesthesia care unit (PACU).Open abdominal surgery usually refers to surgery performed through larger surgical incisions. Closed abdominal surgery refers to surgery performed through smaller incisions or lumens.

### Statistical analyses

Establish an Excel spreadsheet to record the original data of the study, which will be double-checked by two individuals. All data were statistically processed using SPSS 20.0. Measurement data were tested for normality. Measurement data conforming to a Normally distributed data are expressed as the mean ± standard deviation (mean ± SD). The comparison between the two groups was performed by the 2-independent sample t test.

Measurement data that did not conform to the normal distribution are presented as the median and interquartile range [M(Q)], and the comparison between groups was performed by the Mann–Whitney U test or Kruskal–Wallis H test. Count data were presented as n (%), and the χ2 test was used for comparison between groups. Significant level α = 0.05.

## Results

**Fig. 1:**
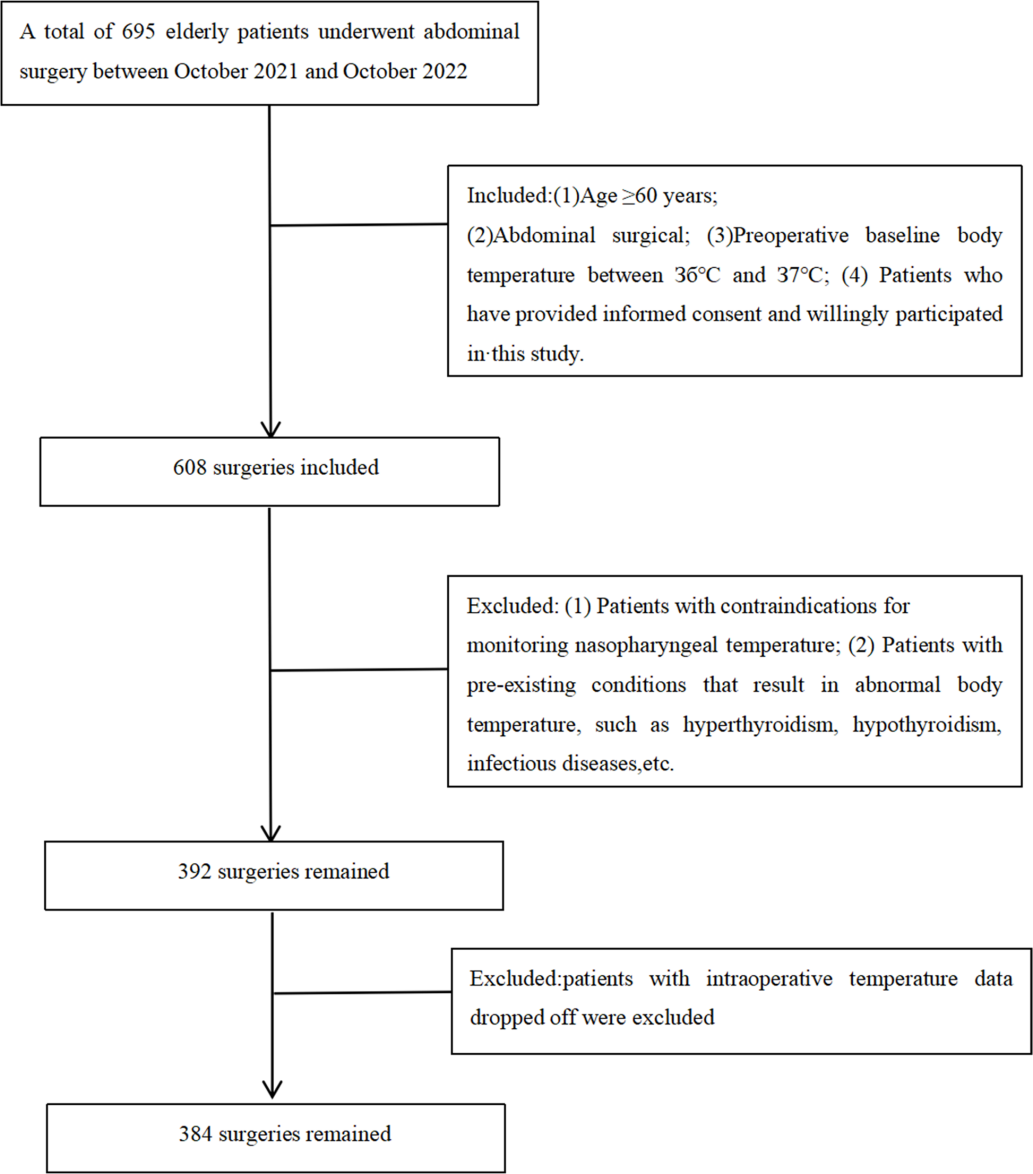
Flowchart of the study summarizing the steps used for patient selection

**Fig. 2:**
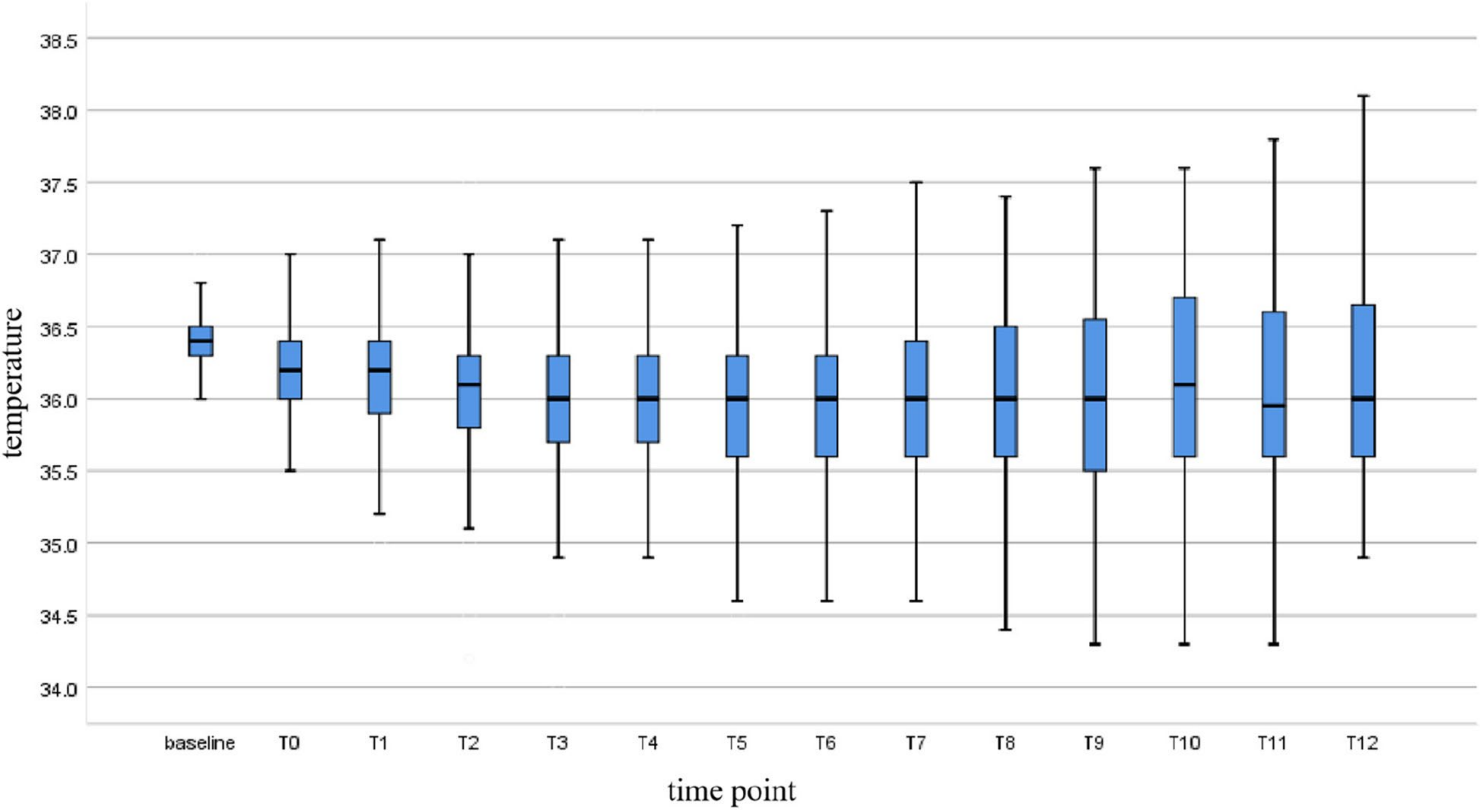
Trends in mean core body temperature of patients at different time points. T0: at the end of anesthesia induction; T1: 0.5h after anesthesia; T2: 1h after anesthesia; T3: 1.5h after anesthesia; T4: 2h after anesthesia; T5: 2.5h after anesthesia; T6: 3h after anesthesia; T7: 3.5h after anesthesia; T8: 4h after anesthesia; T9: 4.5h after anesthesia; T10: 5h after anesthesia; T11: 5.5h after anesthesia; T12: 6h after anesthesia

The data were collected from 384 elderly patients between October 2021 and October 2022 (Fig. 1).A total of 240 patients were in the hypothermia group, while 144 patients were in the nonhypothermia group. The incidence of intraoperative hypothermia was 62.5% among the 384 elderly patients who underwent abdominal surgery, all of whom had mild hypothermia. The average core body temperature of elderly patients undergoing abdominal surgery at all time periods after active intraoperative warming showed a decreasing trend compared with the average preoperative basal body temperature, it decreased to a minimum of 35.96°C at 2h after anesthesia (T4), and then began to rise slowly, but all of them were lower than the patient's average preoperative basal body temperature, as shown in Fig. 2. Table 1 summarizes the demographics of the two groups of patients, with no significant differences in sex, age,body mass index (BMI), duration of surgery, ASA status,urine output between groups,There was statistical difference in the amount of intraoperative infusion.

**Table 1:** Characteristics of the patient population

Indicators	Hypothermia group (*N* = 240)	Nonhypothermia group (*N* = 144)	t/z/χ^2^	p
Sex [N(%)]			0.050	0.911
Male	159(62.1)	97(37.9)		
Female	81(63.3)	47(36.7)		
Age(y)	69.1 ± 5.822	68.59 ± 5.288	-0.880	0.379
BMI [N(%)]			2.993	0.224
< 18.5(kg/m^2^)	15(75)	5(25)		
18.5–23.9(kg/m^2^)	149(64.2)	83(35.8)		
≥ 24(kg/m^2^)	76(57.6)	56(42.4)		
ASA [N(%)]			0.12	0.73
< III	136(63.3)	79(36.7)		
≥ III	104(61.5)	65(38.5)		
Operation time(min)	199.69 ± 78.44	188.94 ± 69.72	-1.354	0.177
Total amount of fluid administration[N(%)]			7.504	0.006
< 1500ml	36(15.0)	38(26.4)		
≥ 1500ml	204(85.0)	106(73.6)		
Urine output (ml)	470.15 ± 293.01	438.79 ± 329.36	-0.961	0.337
Blood loss (ml)	100(50,200)	100(50,300)	-0.748	0.455
Blood transfusion [N(%)]	21(8.8)	16(11.1)	0.699	0.403
Surgical modality [N(%)]			0.048	0.826
Opened surgery	127(52.9)	78(54.2)		
Laparoscopic surgery	113(47.1)	66(45.8)		
Types of surgery			2.141	0.343
Hepatobiliary Surgery	112(46.7)	59(41.0)		
Gastrointestinal Surgery	118(49.1)	75(52.1)		
Pancreas and spleen surgery	10(4.2)	10(6.9)		

Compared to nonhypothermic patients, hypothermic patients had a higher incidence of shivering and significantly prolonged anesthesia recovery time (P < 0.05). There were no statistically significant differences in the occurrence of other adverse events or unwanted events during anesthesia recovery periods (*P* > 0.05) (Table 2). Rates of postoperative wound infection and postoperative hospitalization days were comparable between the hypothermia group and the nonhypothermia group (*P* > 0.05) (Table 3).

**Table 2:** Incidence of adverse events or unwanted events during the anesthesia recovery period

Indicators	hypothermia group (*N* = 240)	nonhypothermia group (*N* = 144)	t/z/χ^2^	P
PACU hypoxemia [N(%)]	74(30.8)	46(31.9)	0.052	0.820
PACU nausea or vomiting[N(%)]	15(6.3)	11(7.6)	0.275	0.600
PACU shivering [N(%)]	33(13.8)	9(6.3)	5.197	0.023
PACU hypertension[N(%)]	44(18.3)	17(11.8)	2.870	0.112
PACU hypokalemia [N(%)]	3(1.3)	5(3.5)	2.179	0.140
PACU hypocalcemia [N(%)]	10(4.2)	7(4.9)	0.103	0.749
PACU analgesic drug use [N(%)]	55(22.9)	23(16.0)	2.681	0.102
anesthesia recovery time(min)	98.75 ± 36.26	90.19 ± 34.91	-2.269	0.02

**Table 3:** Postoperative hospitalization days and postoperative wound infection rate

Indicators	hypothermia group (*N* = 240)	nonhypothermia group (*N* = 144)	t/z/χ^2^	P
Postoperative hospitalization days (d)	6.90 ± 3.52	6.99 ± 3.90	0.224	0.823
postoperative wound infection [N(%)]	10(4.2)	6(4.1)	0.000	0.596

## Discussion

The study comprised 384 patients, and the findings revealed that following active intraoperative warming, there were no occurrences of moderate or severe hypothermia. Nevertheless, The prevalence of mild low hypothermia remained relatively high. This study provides further evidence for the importance of utilizing an inflatable warming blanket for active intraoperative warming in elderly patients undergoing abdominal surgery. It has been shown to effectively prevent the occurrence of moderate and severe hypothermia during surgery. However, the incidence of mild hypothermia remains relatively high, indicating the need for additional measures to further reduce its occurrence in future surgeries. Although inflatable warming blankets are currently the most commonly used method of active intraoperative warming. In clinical practice, they have been shown to reduce the loss of skin heat effectively [10]. However, It is important to note that despite the use of active warming, most patients still experience significant temperature redistribution and a decreased core body temperature after anesthesia induction [11, 12]. This effect is particularly pronounced in elderly patients. In this study, the incidence of hypothermia after active intraoperative warming in elderly patients undergoing abdominal surgery was similar to the results of previous studies by Bo Yu-Mei [13] (66.49%) and Alexander [14] (64.3%),but higher than Dong Tao [4] et al. (39.26%). The reasons for this difference may be related to regional variations and differences in the selected sites for temperature monitoring. In this study, the nasopharynx was chosen as the site for monitoring core body temperature. Core body temperature refers to the temperature of the blood and deep organs in the body. The nasopharynx and esophageal temperatures are considered to be the most accurate indicators of core body temperature due to their proximity to major blood vessels and the heart, and the monitoring values are highly reliable [15]. In the study conducted by Dong Tao et al. [4], the monitoring site for core body temperature was the tympanic membrane, which may explain the higher incidence of hypothermia during surgery in elderly patients in our study.

The results of this study demonstrated that the incidence of shivering during the anesthesia recovery period was higher in the hypothermia group than in the nonhypothermia group, and the difference in incidence between the two groups was statistically significant (*P* < 0.05), which is consistent with the findings of Yi [16] et al. Eberhart LH [17] et al. also identified intraoperative hypothermia as one of the risk factors for postoperative shivering in patients. Shivering is a physiological response of the body, characterized by muscle tremors that serve to increase heat production when the patient's core temperature drops. Although shivering does not lead to severe complications such as patient mortality, it can contribute to discomfort, anxiety, fear, and other adverse emotions during the patient's recovery period, thereby impeding postoperative rehabilitation and impacting patient satisfaction during hospitalization [18]. Related studies [19] have also indicated that the occurrence of shivering can increase the body's oxygen consumption and elevate the risk of tissue hypoxia, which is not conducive to early postoperative recovery.

Therefore, healthcare professionals in the operating room should actively monitor and manage patient body temperature during the perioperative period, reducing the occurrence of intraoperative hypothermia in order to prevent shivering during anesthesia recovery, which holds significant clinical significance.

In this study, the difference in the anesthesia recovery time between the hypothermia group and the nonhypothermia group of elderly individual patients undergoing abdominal surgery was observed.The occurrence of mild hypothermia after active warming during surgery in elderly abdominal surgery patients prolonged the anesthesia recovery time,which is consistent with the results of Tanaka M [20] and other international studies. In a study conducted by Zhou Hui [21] and colleagues in China, it was also observed that hypothermia can lead to a decrease in the patient's metabolic rate, a reduction in blood flow through the liver and kidneys per unit of time, an increase in the metabolism time of anesthetic drugs, and consequently, a corresponding prolongation of the patient's anesthesia recovery time. The PACU plays a vital role in the concept of accelerated recovery surgery, providing meticulous monitoring for patients after anesthesia surgery, thereby enhancing the quality of patients who underwent abdominal surgery.

Anesthesia recovery, reducing postoperative complications, and facilitating accelerated recovery after surgery. Shortening the anesthesia recovery time for surgical patients can expedite turn over between surgeries, enhance operating room utilization, and minimize the wastage of medical resources. Therefore, it is essential for the surgical staff to closely monitor the perioperative temperature of elderly patients undergoing abdominal surgery and refine the management measures for preventing intraoperative hypothermia. For patients who experience intraoperative hypothermia, early rewarming measures should be implemented to minimize the impact of hypothermia on the patient's anesthesia recovery time.

Numerous studies have been conducted both domestically and internationally regarding the occurrence of intraoperative hypothermia in elderly patients and its impact on prognosis.

However, there have been limited studies on the occurrence of intraoperative mild hypothermia and its impact on adverse events or unwanted events during anesthesia recovery. The study analyzed the effect of mild hypothermia on the occurrence of adverse events during anesthesia resuscitation in elderly patients undergoing abdominal surgery, suggesting that mild intraoperative hypothermia also increases the incidence of shivering and prolongs the awakening time during anesthesia resuscitation in elderly patients undergoing abdominal surgery, which reminds the clinical staff that the occurrence of mild intraoperative hypothermia in the patients should not be ignored. In addition, we considered more variables than in previous studies. All these factors increased the reliability and validity of our study.

### Limitations

This study was conducted on elderly abdominal surgery patients in a tertiary hospital in Chengdu, Sichuan Province, China. The sample size was limited, which may affect the representativeness of the findings and introduce certain limitations. In this study, all patients experienced mild intraoperative hypothermia, so only the impact of mild hypothermia on adverse events or unwanted events during anesthesia recovery was analyzed. The impact of moderate and severe hypothermia on adverse reactions during anesthesia recovery was not analyzed. Further multicenter studies with larger sample sizes are needed to validate these findings.

## Conclusions

The incidence of intraoperative mild hypothermia after active warming was high in elderly and intraoperative mild hypothermia increased the incidence of shivering and prolonged anesthesia recovery time, but it had no effect on the occurrence of complications such as nausea or vomiting, hypoxemia and hypertension during anesthesia recovery.

## Data Availability

The datasets used and/or analyzed during the current study are available from the corresponding author on. Reasonable request.

## Abbreviations

ASA: American Society of Anesthesiology
PACU: Postanesthesia care unit
BMI: body mass index
